# Creation and validation of a high-fidelity simulation scenario for suicide postvention

**DOI:** 10.1590/1518-8345.6034.3699

**Published:** 2022-10-17

**Authors:** Laysa Fernanda Silva Pedrollo, Aline Conceição Silva, Ana Carolina Guidorizzi Zanetti, Kelly Graziani Giacchero Vedana

**Affiliations:** 1 Universidade de São Paulo, Escola de Enfermagem de Ribeirão Preto, PAHO/WHO Collaborating Centre for Nursing Research Development, Ribeirão Preto, SP, Brazil.; 2 Scholarship holder at the Coordenação de Aperfeiçoamento de Pessoal de Nível Superior (CAPES), Brazil.

**Keywords:** Suicide, Bereavement, Mental Health, High Fidelity Simulation Training, Simulation Training, Patient Simulation, Suicídio, Luto, Saúde Mental, Treinamento com Simulação de Alta Fidelidade, Treinamento por Simulação, Simulação de Paciente, Suicidio, Luto, Salud Mental, Capacitación con Simulación de Alta Fidelidad, Capacitación por Simulación, Simulación de Paciente

## Abstract

**Objective:**

to create and validate a high-fidelity simulation scenario about the initial support to suicide bereaved people.

**Method:**

a methodological research study to create and validate a simulation scenario about postvention. Its creation was based on scientific recommendations, the validation process was carried out by experts, based on an instrument developed by the authors; the data were statistically analyzed using the Content Validity Index and Gwet concordance coefficient.

**Results:**

the scenario was created to provide initial support to suicide bereaved people in the Primary Health Care context. As learning objectives, welcoming, health care and organization monitoring were proposed according to technical-scientific recommendations. The scenario was validated by 10 specialists in the themes of postvention (5 judges) and high-fidelity simulation (5 judges). The scenario items met the acceptance and reliability criteria (Content Validity Index = 0.80) and satisfactory concordance (Gwet coefficient = 0.640).

**Conclusion:**

the study presented in full a scenario on postvention with innovative potential that can be used free of charge in clinical simulation development during training of different categories of health professionals, to act in support of suicide bereaved people.

## Resumo

**Objetivo:**

construir e validar um cenário de simulação de alta fidelidade relacionado ao apoio inicial aos enlutados por suicídio.

**Método:**

pesquisa metodológica de construção e validação de um cenário de simulação sobre posvenção. A construção foi baseada em recomendações científicas e a validação realizada por especialistas, a partir de instrumento elaborado pelas autoras; os dados foram analisados estatisticamente por meio do Índice de Validade de Conteúdo e do coeficiente de concordância de Gwet*.*

**Resultados:**

o cenário foi construído para o apoio inicial ao enlutado por suicídio no contexto da Atenção Primária em Saúde. Foram propostos como objetivos de aprendizagem o acolhimento, o cuidado em saúde e a organização de acompanhamento de acordo com as recomendações técnico-científicas. O cenário foi validado por 10 especialistas nas temáticas posvenção (5 juízes) e simulação de alta fidelidade (5 juízes). Os itens do cenário atenderam aos critérios de aceitação e confiabilidade (Índice de Validade de Conteúdo = 0.80) e satisfatória concordância (coeficiente de Gwet = 0.640).

**Conclusão:**

o estudo apresentou, na íntegra, um cenário sobre a posvenção com potencial inovador, que pode ser empregado gratuitamente no desenvolvimento da simulação clínica, na formação de diferentes categorias profissionais em saúde, para atuarem no apoio aos enlutados por suicídio.

## Resumen

**Objetivo:**

crear y validar un escenario de simulación de alta fidelidad sobre el apoyo inicial a que se le brinda los que están en duelo por suicidio.

**Método:**

investigación metodológica para la creación y validación de un escenario de simulación sobre postvención. La creación se basó en recomendaciones científicas y la validación fue realizada por especialistas, a partir de un instrumento desarrollado por las autoras; los datos fueron analizados estadísticamente mediante el Índice de Validez de Contenido y el coeficiente de concordancia de Gwet.

**Resultados:**

se creó el escenario para el apoyo inicial al que está en duelo por suicidio en el contexto de la Atención Primaria de la Salud. Se propusieron como objetivos de aprendizaje la organización de la recepción, la atención de salud y el seguimiento según las recomendaciones técnico-científicas. El escenario fue validado por 10 especialistas en la temática postvención (5 jueces) y simulación de alta fidelidad (5 jueces). Los ítems del escenario cumplieron con los criterios de aceptación y confiabilidad (Índice de Validez de Contenido = 0,80) y concordancia satisfactoria (coeficiente Gwet = 0,640).

**Conclusión:**

el estudio presentó un escenario totalmente disponible sobre postvención con potencial innovador que puede ser utilizado gratuitamente en el desarrollo de la simulación clínica para capacitar a diferentes categorías profesionales de la salud, para que den apoyo a los que están en duelo por suicidio.

**Highlights**

(1) Simulation scenario about postvention created and validated by specialists.

(2) Unprecedented product, made available in full and with innovating potential.

(3) Elaborated scenario showed reliability and concordance.

(4) It is a simulation proposed for the training of different professional categories in the heath area.

(5) Contribution in the processes to improve care on postvention.

## Introduction

Suicide is a social, complex and multifactorial phenomenon with significant numbers and impact at the global level^1^. A little explored aspect of suicide prevention is postvention, which refers to a wide set of actions and strategies carried out after a death due to suicide with the survivors^2^ ^-^ ^3^. Listed in the scientific literature as an essential factor for suicide prevention in different contexts, postvention is related to care, welcoming, social and individual support provided to a suicide bereaved individual^3^ ^-^ ^5^. 

More than 130 people are directly or indirectly affected by a single death due to suicide^2^. Suicide bereaved people can experience different feelings, sensations, reactions and psychological and physical changes during grief, which can present different aspects of experiences from other types of bereavement^6^ ^-^ ^7^. 

Improving strategies and interventions focused on training and qualification of health human resources for the prevention and postvention of suicide is fundamental to manage the problem^3^ ^,^ ^8^. Thus, clinical simulation is considered a promising educational strategy to qualify human resources in the health area^9^.

Structured and systematized planning of a high-fidelity simulation scenario is fundamental for the simulated clinical practice to be successful^10^ ^-^ ^11^. For this reason, creation of a scenario is the first stage of a carefully developed proposal and must consider aspects of planning, development, reflection and evaluation of the simulation^10^ ^-^ ^13^. Another extremely important aspect is validation of the scenario by expert judges, in order to verify validity of the objectives and results proposed for the simulation^14^.

The lack of Brazilian studies on postvention, as well as of creative and innovative methods for the training of human resources in health based on good care practices, highlights the need to expand discussions and knowledge, focusing on support for suicide bereaved people^8^ ^,^ ^15^. In this context, development of a fully available high-fidelity clinical simulation scenario for postvention enables scientific progress on the theme with potential subsidies and results for health care improvement. Thus, the current study aims to create and validate a high-fidelity simulation scenario about the initial support to suicide bereaved people.

## Method

### Study design

This a research study of a methodological nature^16^ that describes the creation and validation of a high-fidelity clinical simulation scenario about suicide postvention. 

### Creation of the simulated scenario

The scenario was created in a Higher Education Institution in the city of Ribeirão Preto, State of São Paulo, from a script previously prepared by the research authors and in accordance with national and international recommendations on high-fidelity clinical simulation for the training of human resources in health^10^ ^-^ ^11^ ^,^ ^17^. A number of researchers and a Nursing professor specialized in the clinical simulation and mental health areas took part in creation of the scenario. 

The script built for elaboration of the scenario was defined in two sections and structured in 12 items, which direct planning (seven items) and development of a high-fidelity simulation (five items). This division proposal was defined in order to facilitate organization of the diverse information and contents necessary for preparing the simulation, such as the necessary prior knowledge, learning objective, preparation and development of the simulated activity, theoretical foundation and debriefing.

Preparation of the scenario content was grounded on the current national and international literature about the themes of suicide bereavement and postvention^3^ ^-^ ^4^ ^,^ ^8^ ^,^ ^18^ ^-^ ^23^. The scenario was internally evaluated and reviewed by members (undergraduate and graduate students) of the authors’ research team. 

Through internal evaluation, changes were suggested in relation to the scenario content and appearance, in addition to the final review of the material, considering spelling and grammatical aspects of the writing. The changes proposed were discussed among the research authors and, subsequently, they were accepted or refused according to the possibility of improving and adapting the scenario. Based on the adjustments made in the internal evaluation and validation stages, the final version of the scenario was defined, in order to address and emphasize real aspects related to the initial support that has to be provided by students and health professionals to suicide bereaved people.

### Face and content validation of the simulated scenario

The validation process for the scenario was conducted between March and September 2020 by means of online virtual tools. At this stage, it was decided to select 10 expert judges^16^ ^,^ ^24^, five with expertise in postvention and another five with expertise in high-fidelity simulation. 

### Selection of the participants or specialists 

For selection and characterization of the specialists, a number of adapted criteria about expertise in the themes were used^25^. The criteria were having a curriculum on the Lattes Platform (a channel created by the National Council for Scientific and Technological Development -CNPq- that integrates the curricula and centralizes the scientific information of Brazilian researchers) that proves that the specialist meets, at least, one of the following items: a) Master’s or PhD degree with a paper on the topic (postvention and high-fidelity simulation); b) guidance of academic papers on the topic; c) teaching experience in the area; d) authorship of scientific articles on the theme in high-impact journals; e) guest speaker at a national or international scientific event on the theme.

The search for specialists was carried out via Lattes Curriculum, using the “search curriculum” tool with active search filters: search by subject matter; databases (PhDs); nationality (Brazilian); country of nationality (all: automatic option of the system); filters (none), preferences (none) and advanced search (not used). Two different data collection procedures were conducted: the first about postvention and the second about high-fidelity simulation.

The judges selected were invited to participate in the validation stage via email contacts. The initial invitation was sent containing the invitation letter and the access link to the Free and Informed Consent Form (FICF) of the survey in electronic format (*Google Forms*). Those who accepted to participate were sent a new message with a link to access the research online form. Each judge was advised about the 30-day period for returning validation of the material, with a reminder about the response sent 10 days before deadline. The judges who did not respond to the research by the stipulated date were considered as dropouts. In all, 36 invitations to participate were sent, nine invitations to postvention expert judges and 27 to high-fidelity simulation expert judges.

### Instruments 

A questionnaire for sociodemographic characterization of the judges and a scenario validation instrument were used, both prepared by the authors and evaluated by the research group. The sociodemographic questionnaire contained questions for the participants’ personal (gender, age, geographic location) and professional (academic training, professional experience, length of experience, previous contact with the theme of postvention, previous contact with the theme of simulation) characterization.

The scenario validation instrument was proposed for each scenario item through a three-point Likert scale (adequate, fair and inadequate), with a field for filling in suggestions (optional answer). 

### Data treatment and analysis 

The data obtained from the 10 evaluations by the expert judges were organized and transcribed into a *Microsoft Excel 10* spreadsheet, with double typing and crossing of the typings. The study analyses were performed with the statistical support, by means of the *R* software. 

The data obtained in the questionnaire for the sociodemographic characterization of the judges were analyzed by means of descriptive statistics. The statistical analysis of the data regarding validation of the scenario was carried out using the Content Validity Index (CVI) and the Gwet agreement coefficient, the First-order Agreement Coefficient (AC1)^26^ ^-^ ^27^. For the study, CVI values equal to or greater than 0.80 or 80% were considered (obtained from the following calculation: “total number of ‘adequate and fair’ answers/total number of answers”) and AC1 values close to 1 representing greater agreement (< 0.40 poor; from 0.41 to 0.75 satisfactory to good and from 0.75 to 1.00 excellent)^28^.

The compilation of suggestions and comments received in the judges’ evaluations was transcribed into an editable document, ordered according to each item in the template created specifically for this study and reviewed by the research authors, as a basis for necessary changes in the scenario. 

### Ethical aspects

The study was approved by the Research Ethics Committee (*Comitê de Ética em Pesquisa*, CEP) of the locus institution, under opinion No. 3,742,077. All the research stages followed the guidelines and ethical precepts proposed by CNS Resolution 466 of 12/2012. 

## Results

### Validation of the simulated scenario

The postvention scenario was validated by 10 experts, mostly women (90%), with a mean age of 44.6 years old (minimum 31, maximum 58, median 43.5, and standard deviation = 8.02). In relation to the geographical location of the judges, three were from the Southeast region (30%), another three were from the South region (30%), two were from the Northeast region (20%) and another two were from the Midwest region (20%). 

The study participants attended the following undergraduate courses: Nursing (50%), Psychology (40%) and Medicine (10%). The experts developed their professional activities in three areas: Teaching (70%), Clinical Psychology (20%) and Medicine with an emphasis on Psychiatry (10%). In relation to the time of professional performance, the mean was 19.5 years (minimum of 10, maximum of 30, median of 20 and standard deviation = 7.1).

In relation to acceptance and agreement (CVI), all the scenario items obtained values equal to or greater than 0.90, thus reaching the minimum approval criterion (CVI = 0.80) (Table 1). The Yes (adequate and fair) and No (inadequate) options were used for both analyses.

**Table 1:** Content Validity Index (CVI*) corresponding to validation of the high-fidelity simulation scenario about postvention with expert judges (n^†^=10). Ribeirão Preto, SP, Brazil, 2020

Item	Items		CVI^*^
	Yes n^†^	No n^†^	
Title	10	-	1.00
Objective	10	-	1.00
Target audience	9	1	0.9
Number of participants	9	1	0.9
Physical and material resources	9	1	0.9
Previous study	9	1	0.9
Duration	10	-	1.00
Pre-briefing (contracts and conduction)	10	-	1.00
Pre-briefing (guidelines)	10	-	1.00
Instructions for the simulated patient	10	-	1.00
OSCE^‡^	10	-	1.00
OSCE^‡^ 1	10	-	1.00
OSCE^‡^ 2	9	1	0.9
OSCE^‡^ 3	10	-	1.00
OSCE^‡^ 4	10	-	1.00
OSCE^‡^ 5	10	-	1.00
OSCE^‡^ 6	10	-	1.00
OSCE^‡^ 7	10	-	1.00
OSCE^‡^ 8	10	-	1.00
OSCE^‡^ 9	10	-	1.00
OSCE^‡^ 10	10	-	1.00
Debriefing - Descriptive Phase	10	-	1.00
Debriefing - Analytical Phase	10	-	1.00
Debriefing - Application Phase	10	-	1.00
References	10	-	1.00

* CVI = Content Validity Index; ^†^n = Number of participants; ^‡^OSCE = Objective Structured Clinical Examination

Regarding agreement, the postvention simulation scenario presented results considered from satisfactory to good, with emphasis on the value obtained in the analysis referring to the high-fidelity simulation judges (AC1: 0.743; SD: 0.071; CI: 0.595-0.890) (Table 2).

**Table 2:** Gwet’s AC1^*^ coefficient of validation corresponding to the high-fidelity simulation scenario about postvention with expert judges (n^†^=10). Ribeirão Preto, SP, Brazil, 2020

	n^†^	AC1^*^	SD^‡^	CI^§^
General	10	0.640	0.060	0.515-0.764
Postvention	05	0.499	0.073	0.347-0.650
High-fidelity simulation	05	0.743	0.071	0.595-0.890

* AC1 = Gwet’s AC1 First-order Agreement Coefficient; ^†^n = Number of expert judges; ^‡^SD = Standard Deviation; ^§^CI = 95% Confidence Interval

The simulated scenario about postvention was improved from small changes implemented based on the specialists’ considerations. The changes made included definition of the scenario target audience (undergraduate students and health professionals with previous experience on the theme); standardization of the term “simulated patient”; detailing of the material resources used in the simulation; addition of the item “partially” in the Objective Structured Clinical Examination (OSCE) items and inclusion of the theoretical framework used in the debriefing.

The final and validated version (freely translated into English) of the scenario entitled “Initial support for suicide bereaved people (postvention)” is made available in the sequence (Figure 1). 

**Figure 1:** Final version validated by expert judges (n=10) of the high-fidelity clinical simulation scenario about postvention. Ribeirão Preto, SP, Brazil, 2020

*Title of the scenario:* Initial support for suicide bereaved people (postvention)	
*General objective:* To develop initial support actions to suicide bereaved people during a home visit.	
*Target audience of the scenario (scenario participants)*: Undergraduate students from the health area (having attended some academic discipline related to Mental Health/Psychiatry) and health professionals.	
*Number of people required to develop the scenario:* • Two simulation facilitators (in charge of planning, coordinating and developing the simulated activity); • Two participants (target audience), who took part in the simulated activity; • One simulated patient (who will simulate the person treated in the scenario); • Observers (other participants from the target audience who will externally monitor development of the scenario proposed, as observers of the simulation).	
*Physical and material resources:* • Physical: Teaching or educational practice laboratory, that simulates a living room from a house (depending on the regional context) to conduct a home visit. • Materials: Common objects that make up a domestic environment (living room) depending on the regional context, such as chairs, cushions, rocking hammock, radio or television, glasses, books, pens and decorative objects, among others.	
*Materials for prior study by participants and observers (provided by the scenario coordinators, via email, for the participants and observers to read in advance):* • Previous reading of the booklet entitled “*Lidando com o Luto por Suicídio*” (“Leading with Suicide Bereavement”). Available in: https://inspiracao-leps.com.br/cartilhas-e-e-books/lidando-com-o-luto/ • Audiovisual support material about the theme of postvention: Interview called “September is the suicide prevention month”, conducted by *Rádio Universidade de São Paulo* (2018) with PhD Professor Kelly Graziani Giacchero Vedana. Available in: https://jornal.usp.br/atualidades/setembro-e-o-mes-de-prevencao-ao-suicidio/	
*Estimated duration for each of the scenario stages:* 1. Pre-briefing (15 minutes); 2. Simulation (20 minutes); 3. Debriefing (40 minutes).	
*Pre-briefing (diverse information about contracts and conduction of the simulation):* 1. Introduce the environment to the scenario participants; 2. Discuss contracts about emotional safety: secrecy, anonymity, respect and importance of participating in the discussion after the simulation. 3. The following is not foreseen for this simulated case: handoff, reading the user’s medical chart and presentation and/or use of drug prescription.	
*Pre-briefing (basic guidelines for the simulated case - They can be read and no information should be omitted):* This simulation will be developed with the participation of a simulated patient. You are health students/professionals and are in a Basic Health Unit. For today’s activity, you were requested by the health team to carry out a home visit to Mrs. Marta, 44, who lost her son Bruno, 22, due to suicide 3 days ago. You have approximately 20 minutes to perform the initial welcoming of Mrs. Marta, as you need to return to the health service for a team meeting that will discuss users’ cases. Consequently, it is necessary to evaluate the user’s initial needs and implement the required immediate actions. You have to concentrate on the initial welcoming and support actions towards the user in this case (which are important for the individualized therapeutic plan). Immediate postvention support has already been carried out with the user immediately after the death due to suicide and there will be a subsequent follow-up of the user by the Health Unit, which does not need to be fully planned during the scenario. The simulation laboratory will not be subjected to the intervention of people outside the activity, and will be completed by the simulation facilitators when at least one person from the health team leaves the user’s home or at the end of the maximum execution time. Question for the participants and observers: Do you have any doubt about the guidelines and preparation presented?	
*Instructions for the simulated patient (preparation must be done in the days before the simulation):* You will be Mrs. Martha, 44, who lost her 22-year-old son due to suicide 3 days ago. During the simulation, you should address some feelings, sensations and difficulties experienced in the period of mourning, presented in the form of clues, such as: *Clues that you will necessarily address in the case:* • Unbearable pain and sorrow; • Guilt: “I feel guilty for his death”/“I am to blame for his death, I should’ve done something”; • Anger: “He didn’t think about me, he didn’t think that I’d be left alone”; • Shame: “I don’t want to go out of the house any more, people keep saying things about me”; • Denial and questions related to the death; • “I want to disappear, but not to kill myself”; • Difficulty performing daily activities (self-care); • “People close to me don’t mention my son’s name and don’t want to talk about what happened”. *Clues that you will address if you have the possibility/opportunity to do so:* • Loneliness and isolation; • Lack of listening and attention from other people; • “The wish to sell my house and move”; • Reporting that the son’s birthday would be next month (birthday-related reactions): “I don’t think I can live that day without him!”; • Reporting that she saw some posts by her son some years ago on the Internet about wanting to die, but she thought he was only kidding with friends. *Note:* It is necessary for the simulated patient to know the *“Structured Objective Clinical Examination”* (item below) before the staging, in order to program her clues according to what is expected in the scenario.	
*Objective Structured Clinical Examination* (OSCE^*^). *For each item below, evaluate if the action was performed correctly, using the YES, IN PART or NO options.*	
*Items evaluated*	*Assessment*
OSCE^*^ 1: Enable conversation and listening spaces for bereaved people to recognize and express their feelings, experiences and needs, at their own pace and time.	( ) Yes ( ) In part ( ) No
OSCE^*^ 2: Talk about specifics of suicide bereavement that can generate distress (such as guilt, anger, denial, questioning, loneliness, lack of listening, shame, difficulty approaching what happened, birthday reactions and reductionist explanations about suicide).	( ) Yes ( ) In part ( ) No
OSCE^*^ 3: Encourage bereaved people to express the need for help and the way in which they want to be helped.	( ) Yes ( ) In part ( ) No
OSCE^*^ 4: Identify if the bereaved have aid and support to experience mourning, such as a support network.	( ) Yes ( ) In part ( ) No
OSCE^*^ 5: Guide the bereaved person to seek places and people that make them feel safe and protected, strengthening these supportive relationships in order to avoid isolation due to bereavement (such as family, friends, groups, support groups, among others).	( ) Yes ( ) In part ( ) No
OSCE^*^ 6: Evaluate the presence of suicidal and/or imitative behaviors in the bereaved.	( ) Yes ( ) In part ( ) No
OSCE^*^ 7: Encouraged the bereaved to perform self-care and daily activities, by maintaining a healthy routine that promotes well-being.	( ) Yes ( ) In part ( ) No
OSCE^*^ 8: Instruct the bereaved to avoid making shocking or drastic decisions during mourning.	( ) Yes ( ) In part ( ) No
OSCE^*^ 9: Avoid telling the person what they need to do, what to say or how to feel.	( ) Yes ( ) In part ( ) No
OSCE^*^ 10: Develop empathetic and judgment-free listening during the entire initial support provided to the bereaved.	( ) Yes ( ) In part ( ) No
*Debriefing based on “The Diamond” model (stage developed after the scenario through three consecutive phases)*	
*Descriptive Phase (Evidence perspectives about what happened in the case, without judging the participants’ performance during simulation):* • What happened while offering the initial support to Mrs. Martha? (Question directed to the scenario participants and observers).	
*Analytical Phase (Evidence perspectives about non-technical skills involved in the simulation that were important for the participants):* • How did you feel while offering the initial support to Mrs. Martha? Comment. (Question directed to the scenario participants and observers). • How did you offer the initial support to Mrs. Martha? (Question directed to the observers). • How do you assess your performance in the group work while offering the initial support to Mrs. Martha? (Question directed to the scenario participants). • What positive actions were performed while offering the initial support to Mrs. Martha? (Question directed to the scenario participants and observers).	
*Application Phase (Evidence perspectives about how the participants may apply this knowledge in their clinical practice):* • What would you do differently when facing a new experience of initial support to a suicide bereaved person? (Question directed to the scenario participants). • What can you learn from this experience in the simulation about postvention, and apply it to you professional practice? (Question directed to the scenario participants and observers).	

*OSCE = Objective Structured Clinical Examination

## Discussion

The use of clinical simulation in the training and qualification processes of human resources in health has been the focus of several studies over the last few years^29^ ^-^ ^32^. The initiative to create and validate a high-fidelity simulation scenario related to initial support for suicide bereaved people originated from the interest in grounding knowledge and care processes on postvention, from a realistic approach and with training potential^29^ ^,^ ^31^.

In mental health education, especially in approaches to suicide prevention and postvention, the use of high-fidelity simulation, as well as other innovative methods, is still poorly portrayed at national and international levels^8^ ^,^ ^30^. These findings corroborate the analyses of recent studies showing the difficulties encountered by health professionals in the construction of knowledge and attitudes towards mental health care^33^ ^-^ ^34^.

Development of a clinical simulation is initiated in the structured and systematized creation of a simulated scenario, with a clear definition of the objectives and expected results^35^. The scientific literature about the elaboration of scenarios highlights the importance of resorting to a simulation script that structures the activity to be performed^10^ ^-^ ^11^. 

Among some aspects that comprise elaboration of a scenario, the establishment of theoretical frameworks on the themes of study is evidenced, as well as a careful assessment of needs to be addressed, selection and preparation of the simulated patient, and definition of target audience and necessary resources^10^ ^-^ ^11^ ^,^ ^14^. In addition to that, other issues are important in this process, among them the participation of a prepared and experienced facilitator to conduct the simulated scenario, as well as offering moments of reflection and learning of the practice experienced^10^ ^-^ ^11^ ^,^ ^17^ ^,^ ^36^. 

The scenario entitled “Initial support for suicide bereaved people (postvention)” was developed based on national and international recommendations on clinical simulation and structured in two sections, based on a template mentioned before^10^ ^-^ ^11^ ^,^ ^17^. The objectives and expected results in a high-fidelity simulation, preparation (*pre-briefing*), development (simulation) and reflection for learning (*debriefing*), were included in this construction in order to favor the participants’ teaching-learning process. 

In the *pre-briefing*, guidelines and basic information necessary for the development of the simulated case are presented to the participants, including all preparation prior to the activity^12^ ^,^ ^37^. During simulation development, operational issues are put into practice with the active inclusion of the participants and the simulated patient in the story developed, with the support of facilitators^10^ ^,^ ^38^. The end of the simulated practice is established with a focus on the debriefing, characterized as an important moment of communication with emphasis on reflection, feedback and self-analysis of the participant, mediated in a structured way by the facilitator^13^ ^,^ ^17^. 

The initial support to a suicide bereaved person proposed in this scenario was described as high-fidelity, in order to value the proximity degree of the activity developed with the care reality, including the complexity to be worked on in the scenario^39^. It is worth mentioning that fidelity is related to simulation planning, as realism is based on definitions related to the expected objectives, environment and preparation, choice of participants and physical and material resources to be used^40^.

Characteristics commonly described in the scientific literature on bereavement due to suicide experiences, such as guilt, stigmatization, rejection, shame, anger, less self-care and higher suicide risk, were some of the aspects addressed in the scenario^6^ ^-^ ^7^. The role of health professionals in the support provided is highlighted in this proposal, as postvention approaches are rarely portrayed in the scientific literature, which reinforces the stigma towards the theme and the difficulties approaching suicide^38^.

Studies on interventions carried out with people who have experienced suicide bereavement emphasize the importance of seeking qualified professional help to cope with the loss; however, they highlight the difficulty receiving support^18^. When performed early in time, quickly, actively and through professional actions, postvention presents positive results, with emphasis on welcoming, improvement in well-being and reduction of symptoms related to this process^3^ ^,^ ^41^. In this scenario, the importance of networking is highlighted, with Primary Health Care (PHC) as a gateway to approach the population, especially in carrying out home visits, which is a favorable moment to propose care, especially with regard to postvention^19^. 

It is for this reason that working with high-fidelity simulations can be beneficial for the processes and approaches related to postvention. By providing participant-centered learning experiences, the simulation allows for the performance and experience of a simulated case in an environment developed in a safe, responsible and ethical way, focusing on decision-making, judgment and clinical reasoning, considering innovation and interactivity aspects^40^ ^,^ ^42^.

In this sense, the simulation participants have the opportunity to develop and build, both individually and in groups, theoretical-practical knowledge about postvention from different perspectives of performance in the simulated activity. A number of review studies emphasize that clinical simulation has potential to develop the participants’ attitudes, skills and competencies in the mental health area, even with regard to approaches related to death, especially in training of health professionals for skills involving communication^30^ ^,^ ^43^. 

By presenting characteristics that corroborate with teaching-learning processes, high-fidelity simulation has been characterized as an important link in theoretical-practical approaches to various themes in the health area^29^ ^,^ ^31^ ^-^ ^32^. Among the gains perceived through the simulation, positive and strengthening aspects related to the participants learning stand out, with emphasis on communication, satisfaction, safety between patient-professional, innovation, teamwork and appreciation of the learning process, based on knowledge and critical thinking^30^ ^,^ ^32^ ^,^ ^42^. 

The lack of Brazilian studies on postvention and creative and innovative methods for teaching this theme also highlights the need to deepen the discussions and knowledge with a focus on support for suicide bereaved people^8^. Even with emphasis on the advantages present in professional training involving clinical simulation, the scientific gaps are extensive, something that can be evidenced by the few studies that address suicide prevention and simulation, as well as the non-existence of studies in the scientific literature describing development of the high-fidelity simulation with a focus on postvention^44^.

The validation stage of the simulated scenario, carried out with specialists in the areas of high-fidelity simulation and postvention, allows analyzing the scenario agreement in relation to its items, considering objectives and results proposed for the simulation^45^. Validation confirms that the scenario created meets reality, needs and diverse scientific evidence on the themes worked on, in order to favor the training processes of human resources in health through scientifically based, validated materials, linked to professional practice and capable of promoting education through participatory and interactive methods^46^. 

The cutoff point defined for the study CVI was 0.80 (80%), a value considered in the scientific literature as a parameter for the agreement analysis of the scenario in relation to its items^16^ ^,^ ^47^. It is worth noting that recent studies which validated clinical scenarios in health also considered this analysis parameter^14^ ^,^ ^47^. 

In the analysis performed, all the scenario items were positively evaluated by the expert judges, obtaining values above the cutoff point defined for the study. Even obtaining the CVI, some items received suggestions from the judges, which were analyzed by the researchers and mostly accepted, providing a final version of the scenario in order to favor the initial support provided by professionals and students in the health area for suicide bereaved people. 

Regarding the Gwet agreement coefficient, the AC1 (*First-order Agreement Coefficient*) statistic was used, which analyzes the agreement between the answers given by the evaluating judges^42^. The data from the analysis of the scenario indicate values inside the interval defined as satisfactory to good (AC1 = 0.640; CI: 0.515-0.764), a factor that shows agreement in the answers listed, reinforcing reliability of the analysis^28^. The option to use the agreement coefficient was determined by researchers as a measure recognized in the scientific literature for its robustness, especially in studies with participation of two or more judges, considering analyses on classification scales presenting two or more categories^27^.

The creation and validation of a simulated postvention scenario proposed in this study aims at contributing to improving the training of human resources in health on the initial support to suicide bereaved people. Deepening of studies involving the postvention approach through theoretical-scientific basis has the potential to improve teaching-learning processes in the mental health area involving and recognizing questions about bereavement due to a suicide experience, in order to support future initiatives in the fields of research and clinical practice that may promote and disseminate the theme of postvention at the national and international level^3^ ^-^ ^4^ ^,^ ^8^. 

The positive meanings present in the high-fidelity simulation and the possibility of elaborating a scenario closer to the reality experienced in the clinical practice and in the care provided to the bereaved person enable the theme of postvention to be worked on effectively in the training of human resources in health through simulation-based education. In this way, the scenario that was elaborated and validated stands out for being an unprecedented, accessible product with innovative potential to contribute to the processes of improving health care in support of suicide bereaved people.

The current study offers subsidies for educational (undergraduate and graduate courses) and health (continuing education, among others) institutions in relation to the postvention approach, a theme still little recognized in the processes of training human resources in health. It is for this reason that future studies which promote evaluating the effects of its use in professional training will be important to better understand application of this scenario. The study also provides findings that can be further developed in future research on clinical simulation in mental health field, including approaches to validation of a roadmap for simulation and validation of the scenario in other countries, considering its foundation in international literature.

Among the limitations, the scenario script was prepared by the authors and was not previously validated by expert judges. The scenario created is an unprecedented production that will need periodic updates in its content, according to scientific advances on the themes that are focus of the study; as the effects of evaluating use of the scenario in professional training is an important aspect for future scientific approaches.

## Conclusion

The current study resulted in the creation and validation of a high-fidelity simulation scenario related to the initial support provided to suicide bereaved people, based on recent national and international scientific literature, on the themes of high-fidelity simulation and postvention. Thus, the article presents, in full, a validated scenario that can be used free of charge for the development of clinical simulations in the training of different professional categories for performance in postvention. The validation process by expert judges presented results that indicate agreement in relation to the analyses.
